# Standardizing and scaling up quality adolescent friendly health services in Tanzania

**DOI:** 10.1186/1471-2458-13-579

**Published:** 2013-06-14

**Authors:** Venkatraman Chandra-Mouli, Elizabeth Mapella, Theopista John, Susannah Gibbs, Christina Hanna, Nagbandja Kampatibe, Paul Bloem

**Affiliations:** 1 Department of Reproductive Health and Research, World Health Organization, 20 Avenue Appia, Geneva 27 CH-1211, Switzerland; 2 Department of Reproductive and Child Health, Ministry of Health and Social Welfare, Post Box 9083, Dar es Salaam, Tanzania; 3 Maternal, Newborn, Child Health and Sexual and Reproductive Health, World Health Organization, P.O. Box 9292, Dar es Salaam, Tanzania; 4 Johns Hopkins Bloomberg School of Public Health, Maryland, USA; 5 Georgetown University Medical School, Washington, USA; 6 Consultant Indépendant, Maison Kampatibe, Agonyevé-Cacaveli BP 30395, Lomé, Togo; 7 Department of Immunization, Vaccines and Biologicals, World Health Organization, 20 Avenue Appia, Geneva 27 CH-1211, Switzerland

**Keywords:** Adolescent friendly health services, Tanzania, Quality standards, Scaling up

## Abstract

**Background:**

Adolescents in Tanzania require health services that respond to their sexual and reproductive health – and other – needs and are delivered in a friendly and nonjudgemental manner. Systematizing and expanding the reach of quality adolescent friendly health service provision is part of the Tanzanian Ministry of Health and Social Welfare's (MOHSW) multi-component strategy to promote and safeguard the health of adolescents.

**Objective:**

We set out to identify the progress made by the MOHSW in achieving the objective it had set in its National Adolescent Health and Development Strategy: 2002–2006, to systematize and extend the reach of Adolescent Friendly Health Services (AFHS) in the country.

**Methods:**

We reviewed plans and reports from the MOHSW and journal articles on AFHS. This was supplemented with several of the authors’ experiences of working to make health services in Tanzania adolescent friendly.

**Results:**

The MOHSW identified four key problems with what was being done to make health services adolescent friendly in the country – firstly, it was not fully aware of the various efforts under way; secondly, there was no standardized definition of AFHS; thirdly, it had received reports that the quality of the AFHS being provided by some organizations was poor; and fourthly, only small numbers of adolescents were being reached by the efforts that were under way. The MOHSW responded to these problems by mapping existing services, developing a standardized definition of AFHS, charting out what needed to be done to improve their quality and expand their coverage, and integrating AFHS within wider policy and strategy documents and programmatic measurement instruments. It has also taken important preparatory steps to stimulate and support implementation.

**Conclusion:**

The MOHSW is aware that the focus of the effort must now shift from the national to the regional, council and local levels. The onus is on regional and council health management teams as well as health facility managers to take the steps needed to ensure that all adolescents in the country obtain the sexual and reproductive health (SRH) services they need, delivered in a friendly and non-judgemental manner. But they cannot do this without substantial and ongoing support.

## Introduction

One in five Tanzanians is an adolescent [1]. While many Tanzanian adolescents grow to adulthood healthy, many others do not and are at risk for a range of health problems, including those related to sexual and reproductive health. A large proportion of adolescents in Tanzania are sexually active, but levels of knowledge about and use of condoms are very low. Studies of adolescents in Tanzania indicate that between 20.9% to 63% of females and 24.6% to 84% of males report being sexually active but the percentage who report ever having used a condom ranges from only 4% to 56% [2-4]. According to the 2004–05 Tanzania Demographic and Health Survey two thirds of women are married before their 20th birthday, and just under a quarter (23%) of girls aged 15–19 have begun childbearing [5]. Early childbearing at this age is associated with a heightened risk of maternal mortality and morbidity. Also of concern are the high rates of STIs including HIV among this age group. Youth aged 15 – 24 account for 60% of the new HIV infections in the country [6].

The health system in Tanzania has risen admirably to the challenge of reducing maternal and childhood mortality in the country. For example, the Multi-Country Evaluation of the Integrated Management of Childhood Illness strategy indicated that in Tanzania the programme led to a 13% reduction in under 5 mortality rates (95% CI: 5% to 21%) [7]. Similar efforts can and should be directed towards improving adolescent health.

One study in northern Tanzania has shown that while many primary school students do in fact access health facilities, relatively few receive information about STIs or HIV from health care providers [8]. A survey of secondary school students in Dar es Salaam found that only 6.3% had visited a reproductive health centre [9]. Health care providers agree that teaching young people about sexual and reproductive health is important, [10] and are concerned that many opportunities to reach adolescents with the sexual and reproductive health information and services that they need are being missed.

Many national and international NGOs are implementing initiatives in Tanzania to respond to the SRH needs and problems of adolescents. The Ministry of Health and Social Welfare of Tanzania (MOHSW) is also fully aware that it needs to build on the work of these actors and to harness their strengths more effectively in orchestrating a concerted nationwide response to making existing health services in the country adolescent friendly. This paper describes the problems and challenges that the MOHSW identified, and the way in which it addressed them with the support of WHO, UNFPA and other partners.

## Methodology

To gather facts and figures for the paper, we drew upon published and unpublished MOHSW, in Tanzania’s plans and reports and on journal papers on Adolescent Friendly Health Services (AFHS) in Tanzania published between 2000 and 2012. We supplemented this with our own experiences of working to make health services in Tanzania adolescent friendly. (Two of the coauthors - Mapella and John - live and work in Tanzania. Three others - Chandra-Mouli, Kampatibe and Bloem - have closely supported the work of the MOHSW on adolescent health, which included several trips to the country).

### Initiatives to make health services adolescent friendly in Tanzania prior to the MOHSW’s stepped up effort

In the 1990s, prior to the national-level stewardship efforts by the MOHSW there were a number of smaller-scale initiatives to implement and evaluate AFHS. MEMA kwa Vijana (MkV) was a multifaceted project in Mwanza initiated in 1998 that provided school-based sexual and reproductive health education, strengthened youth-friendly health services, distributed and promoted condoms, and facilitated community activities to address adolescent sexual and reproductive health issues [11]. The project was piloted in four districts and subsequently scaled up to cover the entire Mwanza region [12]. The health service component of the project involved training health care providers on youth friendly sexual and reproductive health services, and an outreach strategy to reach young people in schools [11]. Although the project did not result in significant reductions in STIs and HIV, [13] young men in the intervention area were more likely to use health care facilities and reported that they found service providers to be helpful and respectful [14]. Hindering factors identified during the implementation of the programme included staff shortages, dissatisfaction of staff with the salaries they received, and inadequate facilities to ensure privacy and confidentiality [12].

Another initiative to strengthen AFHS was implemented by Pathfinder International as a part of the African Youth Alliance (AYA) project. This multi-component project started in 2001 and covered ten districts [15]. The health service provision component of the project included training of doctors and nurses and renovation of health facilities. The friendly staff and the welcoming facilities that resulted from the project were clearly appreciated by young people, and as a result, visits by young people seeking SRH services increased substantially [16].

### Problems identified by the Tanzanian government

Through discussions with staff in Tanzanian and international organizations working on adolescent health, participation in meetings and field visits, the MOHSW identified four main problems that were hindering optimum health service delivery to adolescents.

First, many national and international NGOs were offering AFHS and in some places, donor-funded initiatives to make government clinics adolescent friendly were in place. The MOHSW knew of the interest and activity in this area but it was not fully aware of the details and extent of these efforts.

Second, there was no shared understanding or standardized definition of AFHS in the country. Although many organizations were using the label AFHS, the adolescent friendly attributes of health facilities that were part of these different initiatives varied substantially. This contributed to problems both in assessing the adolescent friendliness of health facilities, and in defining ways to improve their adolescent friendliness.

Thirdly, while studies by MkV (Mwanza region) [14], AYA [17] and others had shown that the some of these health facilities were in fact adolescent-friendly, other assessments had shown that service provision by some of the nominally adolescent friendly health centers was poor [18].

Fourthly, while these initiatives were implemented in some parts of Tanzania, there were many other parts of the country where no such initiatives existed, resulting in overall low availability. Furthermore, even in places where such initiatives existed, they were benefiting very small numbers of adolescents, resulting in low coverage. A key factor contributing to this was that the implementation and monitoring of activities to make health services adolescent friendly were not integrated into the overall work of the MOHSW. They were viewed as ‘add ons’.

### The approach of the Tanzanian government to solving these problems

With the support of WHO, UNFPA and other partners, the MOHSW set out to address the problems it had identified. It used a four-pronged approach – firstly, to identify current AFHS activities; secondly, to standardize the definition of AFHS and disseminate this to all of the actors involved; thirdly, to support health facility managers to improve the quality of health services; and fourthly to expand the application of the quality standards in a phased manner across the country.

To address the first problem (i.e. the lack of awareness of what was being done in the country on AFHS), the MOHSW conducted a mapping exercise to identify all the organizations carrying out efforts to make health services adolescent friendly in the country. The mapping was also used to determine the ways in which health facilities were adolescent friendly and the methods that were being employed to make them as such. It was carried out in 2003 by a Tanzanian consultant and provided clear answers to the Ministry of Health [19].

To address the second problem (i.e. the lack of a standardized definition of AFHS) the MOHSW used a two-step process. Firstly, it brought together various stakeholders from the public sector, the private sector, and international and national NGOs that support adolescent health work, for a one-week consensus building workshop to share their experiences in making health services adolescent friendly and to agree on the attributes of AFHS services in the country. Secondly, using the step-by-step process outlined in WHO’s guidance document [20], the MOHSW developed national quality standards for AFHS (Table 1) [21]. Input, process and output criteria to achieve each standard were defined. Indicators to determine if these criteria had been fulfilled and means of verifying them were identified. In addition the actions required by the three levels of implementation – the national unit leading the initiative, the council health management teams and health facility managers were specified. The draft national quality standards and the accompanying elements of a standards-driven quality improvement process were reviewed by all the relevant stakeholders in 2004, approved and then signed off by the Chief Medical Officer in the MOHSW in 2005 [21].

**Table 1:** Standards for adolescent friendly reproductive health services, Tanzania

	
1	All adolescents are able to obtain sexual and reproductive health information and advice relevant to their needs, circumstances, and stages of development
2	All adolescents are able to obtain sexual and reproductive health services that include preventive, promotive, rehabilitative, and curative services that are appropriate to their needs
3	All adolescents are informed of their rights on sexual and reproductive health information and services whereby these rights are observed by all service providers and significant others
4	Service providers in all delivery points have the required knowledge, skills, and positive attitudes to provide sexual and reproductive health services to adolescents effectively in a friendly manner
5	Policies and management systems are in place in all service delivery points in order to support the provision of adolescent friendly sexual and reproductive health services
6	All service delivery points are organized for the provision of adolescent friendly reproductive health services as perceived by adolescents themselves
7	Mechanisms to enhance community and parental support are in place to ensure that adolescents have access to sexual and reproductive health services

Source: Standards for Adolescent Friendly Reproductive Health Services. MOHSW Tanzania, 2005 [21].

To address the third problem (i.e. of uneven quality of health service provision), the MOHSW began actively supporting the application of the national quality standards. To prepare for this it adapted generic published tools previously developed by WHO, Pathfinder, and others for use in Tanzania to train health workers in responding to adolescents effectively and with sensitivity [22]. Complementary tools were developed to inform and engage families and communities. In addition to tools for health facility staff to use, it developed and tested tools to implement and monitor progress towards achievement of national quality standards through actions at the council health management team level. Council health management team staff who had been involved in the process began to apply these tools in health facilities in their jurisdiction.

To address the fourth problem (i.e. patchy and limited coverage), the MOHSW engaged regional and council health management teams to roll out the implementation of the quality standards. An information pack was developed and sent out to all regional medical officers. In addition, MOHSW staff conducted two-day workshops for them in several regions to explain why the national quality standards had been developed and what contribution they could make to ensure that they were achieved in health facilities under their jurisdiction. Alongside this, the MOHSW established a pool of facilitators to build the capacity of health workers and to support council health management teams in incorporating activities to achieve the quality standards in their work plans and budgets. A series of in-service training workshops was conducted and support was provided to council health management teams to incorporate activities and related costs into their work plans and budgets. Alongside this work at the national level, the MOHSW worked hard to press for inclusion of key implementation and monitoring activities into overall national work plans. For example, the national HIV Programme was urged to include modules on preventing HIV and providing care and support to adolescents with HIV in their training programmes, and discussions were initiated on including indicators on AFHS in the national set of programme monitoring measures.

## Results achieved

The MOHSW made important progress in addressing each of the problems it had identified. Firstly, it became fully aware of what was being done by different players within the country to make health services adolescent friendly. Secondly, a standardized definition of AFHS was developed, agreed upon, and the actions needed to achieve the standards at the national, regional, council, and health facility levels were defined. Thirdly, tools to support implementation and monitoring were developed and capacity to use them built. Finally, regional and council health management teams were briefed on the rationale for developing the standards and the actions they would need to take to ensure that they were achieved, including incorporating activities in work plans and budgets to make health facilities in their jurisdiction adolescent friendly. As a result of all this, making health services adolescent friendly moved from being an issue of interest and concern to a small number of NGO projects, donor-supported districts and facilities to a high profile mainstream action carried out by all levels of the public health system. To further consolidate this, the MOHSW included explicit references to AFHS in key documents such as the National Health Policy [23] and the National Health Sector Strategic Plan III [24]. It also initiated discussion on changes to the national Health Management Information System to ensure that age disaggregated data is gathered in health facilities in order to track service-utilization (and reason for attendance) by this age group.

**Figure 1:**
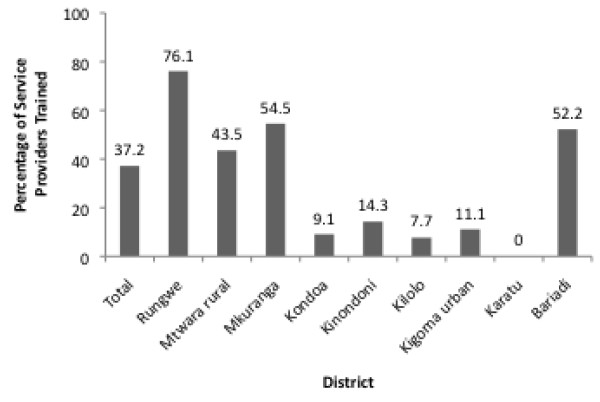
**Percentage of service providers oriented on adolescent sexual and reproductive health information and counseling**[25].

**Figure 2:**
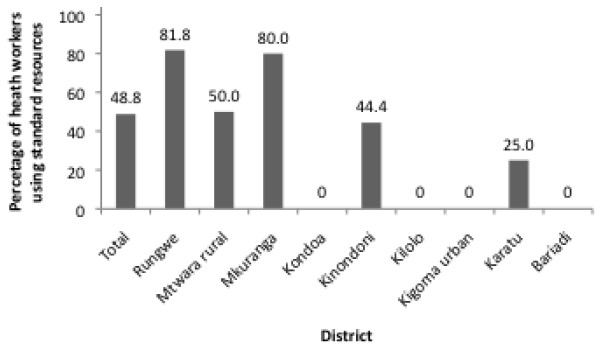
**Percentage of health workers who reported to use standard operating procedures for serving adolescents**[25].

Although important steps were achieved in institutionalizing efforts to make health services adolescent friendly in national, regional and council policies, strategies, work plans and budgets, the effects of this on implementation were mixed. This is evident from the results of an assessment of government health facilities carried out across the country in 2008 to see how they conformed to the national quality standards [25]. The findings reveal very positive results in some areas and relatively poor results in others. For example, standard 4, which stipulates that service providers should have the required knowledge, skills and positive attitudes to provide AFHS, was evaluated through data on the percentage of service providers who had been trained on the provision of AFHS. Data collected on this indicator show that overall 37.2% of the Service Providers (SPs) who were interviewed reported that they had received training in adolescent sexual and reproductive health (ASRH) information and counseling (Figure 1). However, there were disparities in the coverage of orientation and/or training on ASRH information and counseling between the districts. While more than 76% of interviewed SPs in Rungwe district reported to have received training in ASRH information and counseling, none of SPs in Karatu (n=23) had been trained. Standard operating procedures (SOPs), such as the Standards for Adolescent Friendly Reproductive Health Services, were used by only 48.8% service providers in total (Figure 2). However, use of SOPs varied widely by district, with 80% or more using them in Rungwe and Mkuranga and 0% using them in Kondoa, Kilolo, Kigoma urban, and Bariadi. Districts that performed well on service provider trainings were not always the same as the ones that scored well on use of SOPs, underscoring the need to assess the achievement of quality standards using several of the indicators suggested in the Standards for Adolescent Friendly Reproductive Health Services.

**Figure 3:**
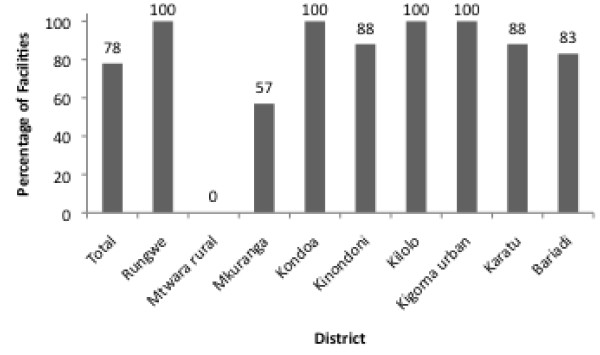
**Percentage of facilities with separate waiting and counseling rooms specifically for adolescents**[25].

**Figure 4:**
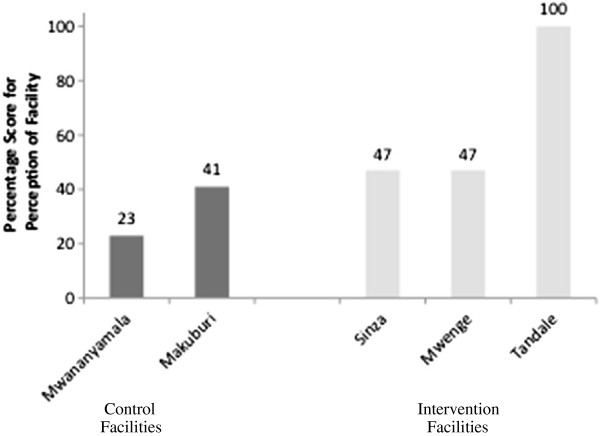
**Adolescent perception of health services, as a percentage score on the criteria for Standard 6 (All service delivery points are organized for the provision of adolescent friendly reproductive health services as perceived by adolescents themselves) – **[26].

One of the criteria used to assess Standard 3, which relates to adolescent sexual and reproductive health rights, is whether providers guarantee privacy and confidentiality when providing adolescents with services. While 78% of facilities in total had separate waiting and counseling rooms specifically for adolescents, some districts lagged behind with 57% in Mkuranga and 0% in Mtwara rural providing separate areas for adolescents (Figure 3). Adolescents’ perceptions of the friendliness of services further confirm inconsistent implementation. Figure 4 presents results from a field-test of the monitoring tools in 2007 [26]. It shows that while facilities in which ameliorative activities had been initiated scored better in the perception of adolescents (Standard 6) than those where they had not, some of them did so only marginally, indicating that there was much scope for improvement. Use of measurable indicators of the AFHS standards has allowed for the identification of health facilities that have done particularly well as well as those that need support in improving services.

Despite the challenges faced in scaling up AFHS, national population-based surveys have indicated that the sexual and reproductive health of adolescents in Tanzania is improving. The age-specific fertility rate for 15–19 year old women has declined from 144 births per 1,000 women in the early 1990s to 116 births per 1,000 women in 2010 [27]. Current modern contraceptive use among women ages 15–19 increased from 5.8% in 1999 to 9.4% in 2010 [28,29]. HIV prevalence declined from 2.1% in 2003/04 to 1.0% in 2007/08 among those aged 15–19 [30,31]. While government efforts related to scaling up AFHS were by no means solely responsible for these improvements in adolescent sexual and reproductive health outcomes, it is certainly possible that the efforts to improve access to and quality of health services for adolescents along with complementary efforts to inform, educate, empower and support adolescents has contributed to improving their health status.

### Lessons learned

One key lesson learned is the positive role of a standardized definition and approach to making health services adolescent friendly. The participatory process to develop standards worked well and was especially effective because it drew upon experience and research in the country to develop a definition that all stakeholders could own and understand.

Another key lesson is that the development and implementation of quality standards is a useful means of ensuring that efforts to make health services adolescent friendly are grounded in wider public health initiatives at the national, regional and council levels. The quality standards not only serve as benchmarks for assessing, guiding, and providing quality adolescent friendly health services, but they are also useful in that they can be institutionalized into work plans and budgets. The Youth Friendly Services training manual [22] developed by the MOHSW as well as the Adolescent Health and Development Strategy [1] supported the scale up of MkV [12] and stimulated financial contribution by some council governments [31]. One concrete example is that following the end of the AYA project several local governments provided funds for the expansion of activities to additional facilities to improve the adolescent friendliness of health facilities [15]. Without nationally incorporated standardized definitions and approaches, the sustainability of such efforts is dependent on the continued availability of funding from donors concerned about these issues.

While randomized trials like MkV have shown some positive results with regard to the efficacy of AFHS and national surveys show improvements in adolescent sexual and reproductive health in Tanzania, data are lacking on the impact of the national scale-up of AFHS. The wider application of the findings of randomized controlled trials such as MkV, which were carried out in a specific context, as well as the challenges of attributing trend data from national surveys to specific policies or programmes call for additional attention to evaluating the impact large-scale interventions [32]. An evaluation platform design that uses districts as the unit of comparison and dose–response analyses is useful for interventions with national coverage and could lead to the identification of attributes of implementation that lead to success in some districts and failure in others [33]. Designing and sustaining an evaluation of this magnitude is costly and would require commitment from the national government as well as funding agencies, but may be worthwhile in order to provide justification for continued allocation of resources to strengthening AFHS.

As results from the MOHSW’s assessment of adolescent sexual and reproductive health services indicate, substantial progress has been made in some districts while others lag behind. Evaluations of the scale up of the health services component of the MkV project to cover the Mwanza region found that contextual factors, such as high turn over rates of local government officials, staff shortages, and inadequately equipped facilities inhibited the scale up of services [12,31]. Similar barriers to scaling up may in part explain why some districts perform better than others.

In order for AFHS to become a reality, more needs to be done to support council health management teams and local health facilities in understanding barriers to implementation and monitoring, and finding creative ways of addressing them. WHO is working with the MOHSW to do just this on three fronts - first, to bring council health management teams together regularly to learn from and support each other in identifying and overcoming barriers to district-level implementation; second, to tap into the expertise that is available in the country - among NGOs, universities and the private sector - to support council health management teams and health facilities; and third, to generate funds to support the scaling up of AFHS.

## Conclusions

The MOHSW has taken on the responsibility for systemizing and scaling up the provision of quality health services to adolescents. It identified four problems that hindered optimum service delivery and addressed each of them. It has made progress in defining what needs to be done at regional and council levels, in communicating this to regional and council health management teams, and in supporting planning and budgeting for this by the latter.

The MOHSW is fully aware that the focus of the effort must now shift from the national to the peripheral levels. It is now working to support regional and council health management teams and health facility managers to take action to reach all adolescents in Tanzania with the health services they need and are entitled to.

## Abbreviations

AFHS: Adolescent Friendly Health Services; AYA: African Youth Alliance; HIV: Human Immuno-Deficiency Virus; MkV: MEMA kwa Vijana; MOHSW: Ministry of Health and Social Welfare; NGO: Non Government Organization; SRH: Sexual and Reproductive Health; STI: Sexually Transmitted Infection; UNFPA: United Nations Population Fund; WHO: World Health Organization.

## Competing interests

We declare that we have no conflicts of interest.

## Authors’ contributions

Chandra-Mouli conceived the paper. With inputs from Hanna, he prepared the first draft. Mapella, Theo, Kampatibe and Bloem provided useful information and inputs to strengthen the paper. Chandra-Mouli worked with Gibbs to revise and finalize the paper. All authors read and approved the final manuscript.

## Authors’ information

Bloem, Chandra-Mouli and John are staff members of the World Health Organization. Kampatibe was a staff member till 2011. He is now an independent consultant. Mapella is a staff member of the Ministry of Health and Social Welfare, Tanzania. Hanna and Gibbs are public health students; both interned with the World Health Organization.

## Pre-publication history

The pre-publication history for this paper can be accessed here:

http://www.biomedcentral.com/1471-2458/13/579/prepub
